# Mechanical, Antibacterial, and Physico-Chemical Properties of Three Different Polymer-Based Direct Restorative Materials: An In Vitro Study

**DOI:** 10.3390/polym17091272

**Published:** 2025-05-06

**Authors:** Chloé Laporte, Rim Bourgi, Hamdi Jmal, Teissir Ben Ammar, Sandy Hazko, Frédéric Addiego, Salvatore Sauro, Youssef Haïkel, Naji Kharouf

**Affiliations:** 1 Department of Biomaterials and Bioengineering, INSERM UMR_S 1121, University of Strasbourg, 67000 Strasbourg, France; chloelaporte9@gmail.com (C.L.); rim.bourgi@hotmail.com (R.B.); tbenammar@unistra.fr (T.B.A.); youssef.haikel@unistra.fr (Y.H.); 2 Department of Endodontics and Conservative Dentistry, Faculty of Dental Medicine, University of Strasbourg, 67000 Strasbourg, France; 3 Pôle de Médecine et Chirurgie Bucco-Dentaire, Hôpital Civil, Hôpitaux Universitaire de Strasbourg, 67000 Strasbourg, France; 4 Department of Restorative Dentistry, School of Dentistry, Saint-Joseph University, Beirut 1107 2180, Lebanon; 5 Mechanics Department, ICube Laboratory, UMR 7357 CNRS, University of Strasbourg, 67000 Strasbourg, France; jmal@unistra.fr; 6 Surgical Department, Medizinische Hochschule Hannover, 30625 Hannover, Germany; sandy.hazko@hotmail.com; 7 Department Materials Research and Technology (MRT), Luxembourg Institute of Science and Technology (LIST), ZAE Robert Steichen, 5 rue Bommel, L-4940 Luxembourg, Luxembourg; frederic.addiego@list.lu; 8 Dental Biomaterials and Minimally Invasive Dentistry, Department of Dentistry, Faculty of Health Sciences, Cardenal Herrera-CEU University, CEU Universities, 46115 Valencia, Spain; salvatore.sauro@uch.ceu.es; 9 Department of Therapeutic Dentistry, I.M. Sechenov First Moscow State Medical University, 119146 Moscow, Russia

**Keywords:** antibacterial activity, compression strength, direct restorative materials, polymer-based, porosity, wettability

## Abstract

A novel resin-based bulk-fill restorative material (ST; Stela SDI, Bayswater, Victoria, Australia) has been recently introduced as a self-curing alternative to traditional light-cured composites. Promoted for its unlimited depth of cure, enhanced aesthetics, and unique primer composition, it aims to address challenges associated with amalgam and light-curing composites. Thus, the aim of this in vitro study was to investigate the performance of the new self-curing polymer-based restorative material, ST, compared to two conventional light-cured composites for direct restoration. The study evaluated compressive strength with and without aging, antibacterial activity, mineral deposition in contact with Phosphate-Buffered Saline (PBS) and artificial saliva, porosity, and wettability of ST (Tetric EvoCeram (TE; Ivoclar Vivadent, Schaan, Liechtenstein) and Clearfil Majesty ES-2 (CM; Kuraray Noritake Dental, Tokyo, Japan)). The data was statistically analyzed (α = 0.05) through one-way and two-way analysis of variance (ANOVA). ST demonstrated significantly higher compressive strength than TE and CM at baseline and after aging (*p* < 0.001), while aging significantly reduced compressive strength across all materials (*p* < 0.001). Fracture mode analysis revealed brittle fractures for TE and CM, whereas ST fractured in multiple smaller fragments. CM showed the highest void volume and diameter, significantly differing from ST and TE (*p* < 0.001). Scanning electron microscopy (SEM) analysis revealed cubical-like crystalline formations on ST’s surface after 28 days of immersion in PBS and saliva, indicating some level of bioactivity, whereas no changes were observed for TE and CM. Wettability testing showed ST had the lowest contact angle (12.24° ± 2.1°) compared to TE (62.78° ± 4.68°) and CM (64.64° ± 3.72°) (*p* < 0.001). Antibacterial activity testing displayed a significant decrease in bacterial growth for CM compared to ST (*p* = 0.001) and TE (*p* = 0.002); however, ST and TE showed no significant differences (*p* = 0.950). To conclude, ST Automix demonstrated promising results across several key parameters, making it a potential candidate for long-lasting restorative applications. Future studies should explore its long-term clinical performance and investigate formulations that enhance its antibacterial properties. Moreover, the bond strength of these materials to dentin and the cytotoxicity should be evaluated.

## 1. Introduction

Dental caries is an infectious disease caused by commensal bacteria such as *Streptococcus mutans*, which ferment dietary carbohydrates to produce acids that demineralize the hard dental tissues (enamel and dentin), leading to cavity formation [1]. Despite a slight decline in prevalence since the 1990s [2], untreated dental caries affected 29% of permanent teeth and 7.8% of primary teeth globally in 2017 [3]. Indeed, dental caries ranked as the most prevalent health condition in the Global Burden of Disease Study published by *The Lancet* in 2019 [4]. Therefore, prevention and treatment of dental caries remain critical public health priorities, as they contribute to significant health disparities worldwide [3]. Conventional treatments of dental caries rely on the removal of affected tissues by employing rotary instruments, followed by reconstruction of the lost dental substances. Until recent years, this restorative phase has been predominantly carried out using dental amalgam in Europe due to its durability and mechanical strength [5]. Nevertheless, advances in adhesive dentistry have led to the widespread use of resin-based composite [6], particularly in developed countries, in which their success was related to their excellent optical and mechanical properties and the lack of requirement for undercut preparation [7]. They were also used in response to the Minamata Convention on the use of mercury [8]. These materials have shown good long-term performance in restoring anterior and posterior cavities and relatively low failure rates after 10–20 years [6,9,10] despite being slightly higher than those of amalgam [5,7].

Composite resins are three-dimensional materials that combine an organic resin matrix with inorganic mineral particles linked by a silane coupling agent. Additionally, composite resins contain polymerization initiators and other agents. However, they do not adhere directly to dental tissues and require an adhesive layer for effective bonding [11]. The use of resin composites is associated with two main shortcomings: polymerization shrinkage and longevity due to hydrolysis of the bonding interface with dental substrates, particularly dentine.

Polymerization shrinkage is an intrinsic property of resinous materials, resulting from the reduction of the volumetric monomer distance during the polymerization reaction, as weak van der Waals forces are converted into covalent bonds [12]. The issue is not the shrinkage itself but the stress generated at the tooth-material interface in a constricted and well-defined space, which can cause enamel cracks, cuspal deflection, microleakage, and postoperative sensitivity. These flaws can lead to restoration debonding and/or secondary caries, making polymerization shrinkage a major cause of resin-based restoration failure [12,13]. Since polymerization shrinkage increases with the volume of the polymerized material, the most effective solution for clinicians to face such a situation is the incremental or layering composite technique. It involves restoring the lost dental substances by applying small increments (1–2 mm) of material, polymerized one after another. Additionally, this technique enhances the degree of conversion of the material, as light scatters more effectively in thinner layers [6,14]. Unfortunately, this technique also has inherent drawbacks, including the fact that it is time-consuming and can potentially lead to bulk defects, such as air bubbles and micro-voids [12]. To address polymerization shrinkage, bulk-fill composites have been developed, allowing for 4–5 mm incremental layers without compromising polymerization performance. This is achieved through the incorporation of additional photoinitiators and enhanced translucency for better light transmission [14]. Although it was demonstrated that this modification reduces polymerization shrinkage, further research is needed to explore this in more depth [12]. Furthermore, it was established that self-curing restorative materials generate less polymerization shrinkage than light-curing materials due to their slower polymerization speed [14]. Specifically, self-cured resin-based composites (RBCs) do not require light exposure as they lack photoinitiators, making them a suitable substitute in case a curing light is unavailable [15,16,17,18,19]. They also have a slower polymerization rate [15] and can achieve a higher degree of conversion when combined with newer monomers and primers [15,16], potentially improving the final restoration quality [17]. A new self-cured bulk-fill resin-based composite (RBC), Stela (ST; SDI, Bayswater, VIC, Australia), has been recently introduced. Available in Automix syringes or capsules, it is marketed as an “amalgam alternative” because it does not require a light-curing unit (LCU), offers unlimited depth of cure, and has superior aesthetics compared to amalgam [20]. The polymerization of ST RBC is initiated when it contacts the ST primer, accelerating the reaction at the tooth/primer-RBC interface. This system uses a novel primer, free from tertiary amines, and includes glycerol-dimethacrylate (GDMA), which may enhance polymerization, mechanical properties, adhesion to dentin, and reduce water uptake and solubility [20]. ST Automix is formulated with various fillers, including strontium fluoroaluminosilicate, ytterbium trifluoride, and calcium aluminate [14]. However, this novel auto-mixed composite material (ST) was rarely studied.

Therefore, the aim of this in vitro study was to evaluate the efficacy of a novel self-curing restoration material, ST Automix, in terms of compressive strength with and without aging, antibacterial activity, mineral deposition in contact with Phosphate-Buffered Saline (PBS), and artificial saliva (AS). The porosity and wettability were also analyzed and compared to two conventional light-curing resin composites. The first null-tested hypothesis was that there would be no difference between ST and the two conventional tested composites in terms of compressive strength and physicochemical properties. The second null hypothesis was that there would be no difference between the tested materials in terms of antibacterial activity.

## 2. Materials and Methods

### 2.1. Materials and Sample Preparations

Three different polymer-based restorative materials: Tetric EvoCeram A3 (TE; Ivoclar Vivadent, Schaan, Liechtenstein), Stela Automix (ST; SDI Ltd., Bayswater, VIC, Australia), and Clearfil Majesty ES-2 A3 (CM; Kuraray Noritake Dental; Tokyo, Japan) were evaluated in this in vitro study (Table 1). In this study, the two conventional light-cured composite materials were used as control groups. These materials were selected based on their established clinical performance, allowing for a comparative assessment against the newly introduced self-curing bulk-fill resin composite, ST. This design facilitated the evaluation of ST’s mechanical, biological, and surface characteristics relative to commonly used benchmark materials. All materials were handled according to the manufacturers’ instructions (Table 1). Standardized cylindrical specimens (3.8 mm in height and 3 mm in diameter) were prepared using custom-made Teflon molds. The molds were placed on a glass plate and filled with each material. For ST, the mold surfaces were pre-coated with Stela Primer (SDI Ltd., Bayswater, VIC, Australia) prior to filling. CM and TE were light-cured for 20 s using an LED curing system (Luxite Lampe LED, ITENA Clinical, Paris, France). ST was left to set chemically for 4 min to ensure complete polymerization.

### 2.2. Compression Strength & Fracture Mode

Forty-five specimens (*n* = 15 per group of polymer-based materials) were stored in distilled water at 37 °C for 24 h and 28 days. Following the immersion periods, a uniaxial compression test was conducted to determine the maximum load without failure of each specimen. A compression test was performed using a universal electromechanical testing device (Instron 3345, Norwood, MA, USA) equipped with a 1 kN load cell (Instron 2519-1 kN) and a displacement sensor. Measurements were carried out at a constant crosshead speed of 0.5 mm/min. The specimens were placed between two lubricated steel plates, with the upper plate positioned as close to the specimen as possible without making contact. A compressive force was then applied until rupture occurred. Force values were recorded using the Bluehill^®^ Universal software, Version 4.03 (Instron, Norwood, MA, USA).

The compressive strength was calculated in megapascals (MPa) according to the following formula:

σc = 4P/πD^2^

where P is the maximum recorded force during testing, and D is the initial sample diameter.

All specimens were then observed under a digital microscope (VHX-5000, Keyence, Osaka, Japan) at 50× magnification to analyze the fracture mode, which was then classified as either brittle or ductile [21]. The mechanical behavior of each material given by the stress-strain curve was also analyzed and compared to the fracture mode.

### 2.3. Porosity

The internal structure of the tested materials was investigated in three-dimensions (3D) through micro-computed X-ray tomography (µCT) (EasyTom 160 from RX Solutions, Chavanod, France). The imaging process was carried out at a voltage of 60 kV and a current of 70 µA, using a micro-focused tube equipped with a tungsten filament. The source-to-detector distance (SDD, 435.5 mm) and the source-to-object distance (SOD, 9.34 mm) were adjusted to achieve a voxel size of approximately 2.72 µm. Volume reconstruction was performed using the software Xact64 (RX Solutions) after adopting geometrical corrections and ring artifact attenuation. 3D image analysis was conducted using the Avizo software 2022-2 (ThermoFisher, Waltham, MA, USA).

### 2.4. Morphological Changes After PBS/Saliva Immersion

Twelve specimens of each material were prepared using the same Teflon molds (3.8 mm in height and 3 mm in diameter) and protocol as before. These specimens were immersed in 10 mL of phosphate-buffered saline (PBS10x, Dominique Dutscher, Bernolsheim, France) or 10 mL of artificial saliva (Pickering Laboratories, Mountain View, CA 94043, USA) at 37 °C. At specified time points—24 h, 7, 14, and 28 days—the specimens were gently washed three times for 5 min in distilled water. All specimens were then sputter-coated with gold-palladium using a Hummer JR sputtering device (Technics, San Jose, CA, USA).

The specimens were then examined using a scanning electron microscope (SEM; Quanta 250 FEG, FEI Company, Eindhoven, The Netherlands) at a magnification of ×2000 to assess morphological changes. The SEM operated with an electron acceleration voltage of 7.5 kV. Additionally, surface chemical analysis was conducted using energy-dispersive X-ray spectroscopy (EDX; Edax AMETEK, San Luis Obispo, CA, USA) at a magnification of ×5000.

### 2.5. Wettability

Three specimens from each material were prepared in Teflon molds (20 mm in diameter and 2 mm in thickness) and used to investigate the sorption time by placing a 4 µL drop of distilled water on the material surface. All the tests were performed at 23°. A contact angle device (Attension Theta, Biolin Scientific, Götenborg, Sweden) was used to measure the interaction. The water droplet’s profile was recorded using a horizontal camera for analysis, and the contact angle was reported after 10 s of depositions.

### 2.6. Antibacterial Activity

*Staphylococcus aureus* (*S. aureus*, ATCC 25923) was chosen for this study due to its clinical relevance as a major pathogenic microorganism in dental infections [1]. The bacteria were cultured according to the manufacturer’s instructions using Muller–Hinton broth “MHB” (Le Pont de Claix, France). In all the following tests, the turbidity of MHB containing *S. aureus* (bacterial medium) was adjusted to OD600 (nm) = 0.3. Three specimens (3.8 mm in height and 3 mm in diameter) from each material were used. Each specimen was placed in contact with 1 mL of the bacterial medium using a 24-well culture plate. In the positive control, the bacterial medium was placed into the well without any material. In the negative control, the well was filled with 21 μL of 20% Chlorohexidine. The plates were then incubated for 24 h at 37 °C under constant stirring (450 rpm). The experiment was performed in triplicates. After each experiment, 50 µL from each well tube was diluted with 50 µL of MHB. The solution was mixed using a vibrator for 10 s, and a microplate absorbance spectrophotometer (xMark^TM^ Biorad, Schiltigheim, France) was used to evaluate the CFU/mL (colony forming units/mL) count.

### 2.7. Statistical Analysis

Statistical analysis was accomplished using SigmaPlot (version 11.2, Systat Software, Inc., San Jose, CA, USA). The means and standard deviations were calculated. Shapiro–Wilk testing was performed to check the normality. One-way analysis of variance on ranks (ANOVA), including a multiple comparison procedure (Tukey testing), was used to determine whether significant differences existed in the antibacterial activity and wettability. A two-way ANOVA was also used to determine whether significant differences existed in terms of compressive strength. Chi-square was used to evaluate the fracture mode. In all the measurements, a statistical significance level α of 0.05 was adopted.

## 3. Results

### 3.1. Compressive Strength and Fracture Mode

**Figure 1:**
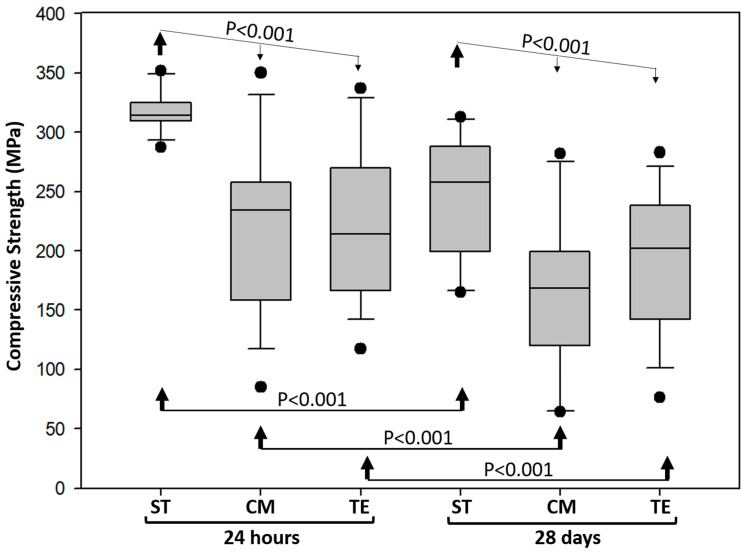
Means and standard deviation values of compressive strength for the three tested materials: ST (Stela Automix), CM (Clearfil Majesty ES-2), and TE (Tetric EvoCeram). Statistical analyses were mentioned by arrows.

Two-way ANOVA revealed a statistically significant effect for both material type and aging time (*p* < 0.001). At both aging time points, ST exhibited a significantly higher compressive strength compared to TE and CM (*p* < 0.001). In contrast, no significant difference was observed between TE and CM (*p* = 0.237) (Figure 1).

Concerning the aging factor, all values obtained with the tested materials demonstrated a significant decrease after 28 days of aging (*p* < 0.001).

**Figure 2:**
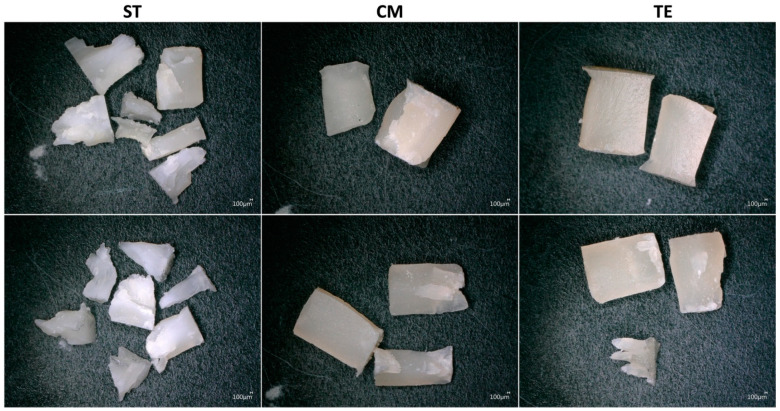
Digital microscopy images demonstrating the fracture of the different materials after the compressive strength test: ST (Stela Automix), CM (Clearfil Majesty ES-2), and TE (Tetric EvoCeram).

After the compression test, all the specimens were observed under a digital microscope to investigate the fracture mode. Bleached and striated zones correspond to areas that underwent plastic deformation and thus, ductile fracture. In contrast, areas that are still transparent without striations showed a rapid, brittle fracture. According to this analysis, CM and TE demonstrated more brittle fracture zones compared to ST, as shown in Figure 2. In addition, it was observed that CM and TE typically fractured into only two or three pieces, whereas ST tended to break into multiple smaller fragments. This illustrates that damage in CM and TE materials is unidirectional and fast, confirming the brittle fracture hypothesis. As for ST, the damage is multidirectional and progressive, resulting in a globally ductile fracture.

### 3.2. Porosity

**Figure 3:**
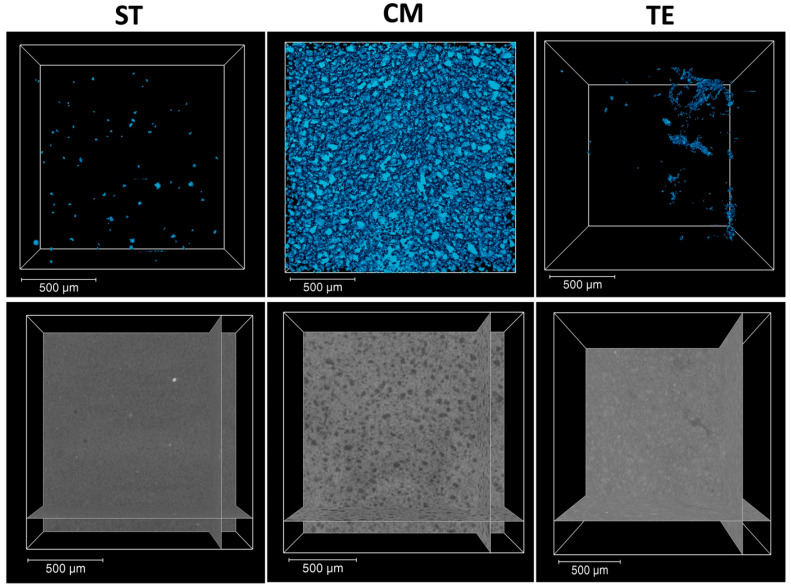
Volume rendering of segmented pores (blue color) with a scale bar of 500 µm, obtained by X-ray tomography analysis in ST (Stela Automix), CM (Clearfil Majesty ES-2), and TE (Tetric EvoCeram).

A higher void volume fraction and larger void diameter were observed for CM (16.8 ± 2.46 µm) compared to ST and TE (Figure 3) (*p* < 0.001). However, no significant difference was found between TE (0.047 ± 0.05 µm) and ST (0.47 ± 0.11 µm) (*p* = 0.864). Accordingly, ST and TE exhibited similar average porosity diameters (18.44 ± 1.2 µm and 15.4 ± 3.55 µm, respectively), which were significantly lower than that of CM (22.81 ± 0.46 µm) (*p* = 0.001).

### 3.3. SEM & EDX Analysis

**Figure 4:**
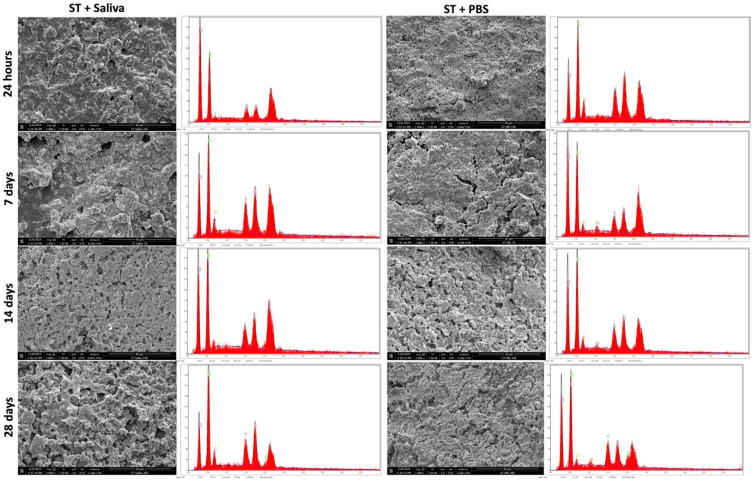
Scanning electron microscope images (2000× magnification) demonstrating the mineral depositions on ST (Stella Automix) after 24 h, 7, 14 and 28 days of immersion in PBS or artificial saliva. EDX analysis demonstrates the chemical compositions of the different surfaces.

**Figure 5:**
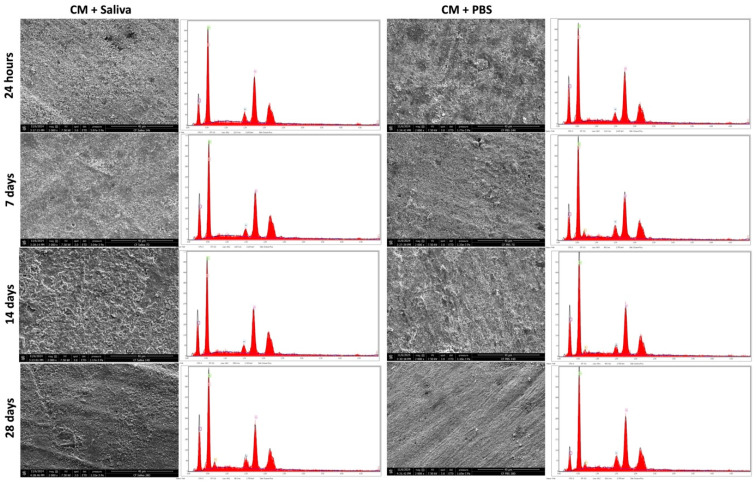
Scanning electron microscope images (2000× magnification) demonstrating the mineral depositions on CM (Clearfil Majesty ES-2) after 24 h, 7, 14 and 28 days of immersion in PBS or artificial saliva. EDX analysis demonstrates the chemical compositions of the different surfaces.

**Figure 6:**
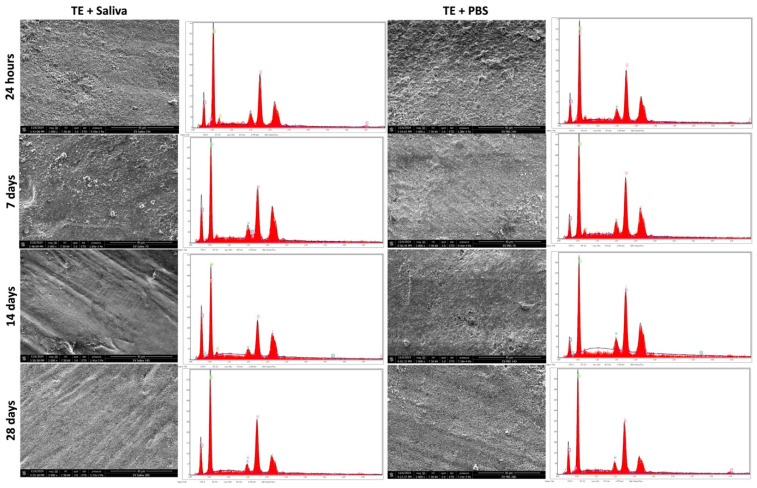
Scanning electron microscope images (2000× magnification) demonstrating the mineral depositions on TE (Tetric EvoCeram) after 24 h, 7, 14, and 28 days of immersion in PBS or artificial saliva. EDX analysis demonstrates the chemical compositions of the different surfaces.

Scanning electron microscopy (SEM) analysis was performed for the three materials after immersion in AS or PBS at different time points. After 28 days of immersion, only ST demonstrated cubical-like crystalline deposition at the surface for both immersion solutions (Figure 4). In contrast, no changes were observed for CM and TE (Figure 5 and Figure 6).

The ability of restorative dental materials to promote mineral deposition and remineralization at the interface between restorations and dental tissue represents an additional characteristic of biomaterials. This property was assessed through energy-dispersive X-ray (EDX) surface analysis, which revealed that while CM and TE specimens exhibited no significant alterations in their surface composition after immersion in PBS or artificial saliva, ST specimens demonstrated evidence of mineral precipitation. These findings were further corroborated by scanning electron microscopy (SEM) analyses, which confirmed the presence of mineral deposits solely on the surface of the ST specimens.

### 3.4. Wettability

**Figure 7:**
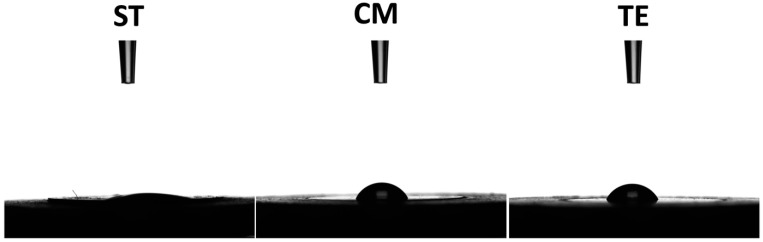
Water drops’ profiles on ST (Stella Automix), CM (Clearfil Majesty ES-2), and TE (Tetric EvoCeram) surfaces after 10 s of water drop deposition.

Our results show that 10s after the water droplet deposition, ST demonstrated a significantly lower contact angle (12.24 ± 2.1°) compared to CM (64.64 ± 3.72°) and TE (62.78 ± 4.68°) (*p* < 0.001) while no significant difference was found between CM and TE (*p* = 0.759) (Figure 7).

### 3.5. Antibacterial Activity

**Figure 8:**
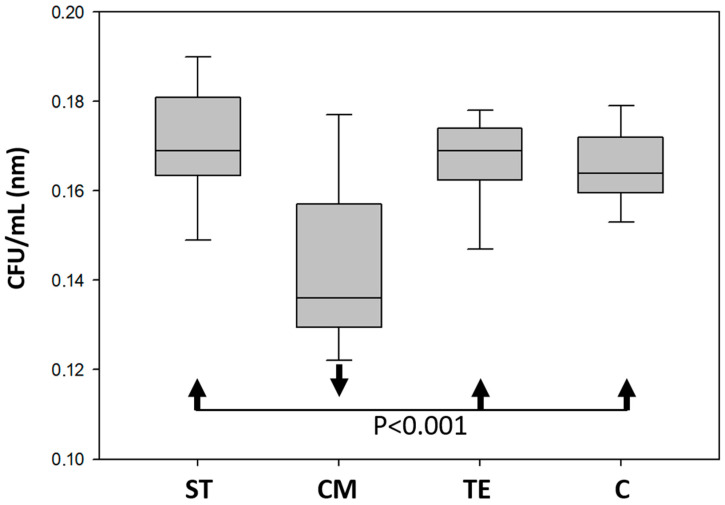
Number of colony-forming units/mL of *Staphylococcus aureus* in contact with ST (Stella Automix), CM (Clearfil Majesty ES-2), TE (Tetric EvoCeram) and C (control group). Statistical analyses were mentioned by arrows.

The antibacterial assessment revealed a statistically significant reduction in bacterial growth in the medium exposed to CM compared to ST (*p* = 0.001), TE (*p* = 0.002), and the control group (*p* = 0.006), suggesting superior antimicrobial properties of CM. However, the antibacterial efficacy showed no statistically significant differences between TE and the control group (*p* = 0.987), ST and TE (*p* = 0.950), or ST and the control group (*p* = 0.819), indicating comparable bacterial growth inhibition patterns among these groups (Figure 8).

**Figure 9:**
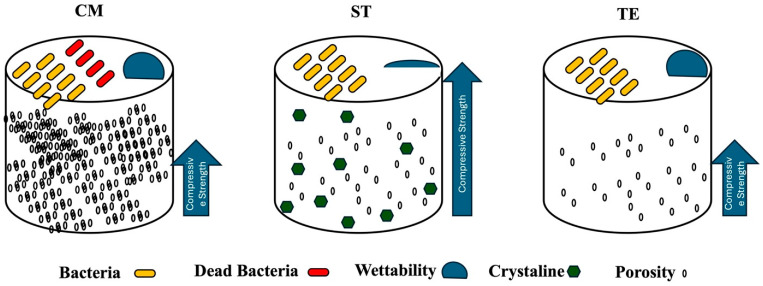
An overview of the project’s main objectives and outcomes with ST (Stella Automix), CM (Clearfil Majesty ES-2), and TE (Tetric EvoCeram).

The project summary is illustrated in Figure 9.

## 4. Discussion

The first null hypothesis, which stated that there is no difference between the tested materials in terms of compressive strength and physicochemical properties, and the second null hypothesis concerning the antibacterial activity were rejected. However, for antibacterial activity, only CM demonstrated a significant effect, while no substantial differences were observed between ST and TE.

ST demonstrated a significantly higher compressive strength compared to TE and CM, both before and after aging. In contrast, TE and CM exhibited comparable compressive strength values but were less resistant to aging-induced degradation.

The enhanced mechanical performance of ST can be attributed to several factors, the first being its distinctive formulation, particularly the inclusion of UDMA, GDMA, and ytterbium trifluoride. Studies have reported that UDMA-based systems generally achieve greater cross-linking density and improved mechanical stability [21,22].

Another key factor contributing to the superior polymerization kinetics of ST is its optimized initiator and co-initiator configuration [20], which promotes more efficient polymer network formation. Although conventional light-cured resins have inherent limitations in polymerization uniformity due to constraints in light penetration, ST’s self-curing mechanism effectively overcomes these challenges. This self-curing capability ensures uniform polymerization throughout the entire matrix material, regardless of light availability, resulting in stronger mechanical properties [23].

The significant reduction in compressive strength observed after aging in all tested materials could be attributed to the expansion of porosities due to storage in an aqueous medium, which increases their volume fraction compared to the initial state, ultimately leading to a decrease in mechanical resistance [24].

The presence of ytterbium trifluoride, along with other components, may have contributed to a greater cross-linking density and improved mechanical stability. In contrast, TE and CM, which rely on Bisphenol A glycidyl methacrylate (Bis-GMA)-based matrices, exhibited comparable compressive strength values but were less resistant to aging-induced degradation [20,23]. This reduced resistance may also be related to the degree of polymerization achieved through the unique composition of the initiator and co-initiator systems [20].

Fracture mode analysis revealed that CM and TE exhibited prevalently brittle fractures with fewer fragments, while ST was characterized by multiple smaller fragments. The ability of a material to resist fracture is closely linked to its internal structure, including the distribution of voids and the strength of the filler-matrix interface. Voids in restorative dental materials can form during the manufacturing process or because of improper handling by clinicians, leading to regions of weakness in the restorations. Brittle fracture patterns, such as those seen in CM and TE, may be linked to the high void fractions and poor filler-matrix adhesion, which allow localized stress concentrations to form under load. These stress concentrations cause rapid crack propagation, leading to fewer and larger fracture fragments. Indeed, this phenomenon was demonstrated in previous studies, where higher void content and larger void diameter were found to significantly contribute to the mechanical failure of resin composites [25]. In contrast, materials like ST, which have a lower void fraction and an improved filler-matrix bonding, demonstrate enhanced fracture toughness. It is possible that the unique integration of the filler-matrix in ST contributed to the distribution of the stress more evenly throughout the material, thus enhancing its fracture resistance. These findings seem to be in accordance with previous reports showing that improved filler-matrix adhesion allows for a better load transfer between the resin matrix and the filler particles, which reduces the chances of crack initiation and propagation [26]. This characteristic contributes to the material’s ability to produce numerous smaller fragments upon fracture as stress is distributed more evenly, subsequently preventing the formation of large cracks. These results support those observed in earlier reports, which suggest that the fracture resistance of composites is closely associated with the void fraction and the filler content [27].

CM exhibited the highest amount of porosity compared to ST and TE. Porosity is a critical factor affecting mechanical properties, as increased void content weakens the structure and increases the risk of cracks [28]. ST seems to have an optimized filler distribution and advanced polymerization kinetics, which likely reduced porosity while contributing to its enhanced mechanical performance [29]. Furthermore, it was indicated that the higher the polymerization shrinkage strain, the lower the amount of porosity [30]. This could explain the fact that the reduced void content in ST was due to more efficient polymerization, which minimized the formation of gaps between the filler particles and the matrix [31]. As the polymerization process progresses, the shrinkage strain can aid in compressing the filler-matrix interface, reducing the occurrence of voids and ensuring a more compact structure. In contrast, materials with a lower polymerization shrinkage, like CM, may have retained more voids, leading to reduced mechanical strength and a higher porosity level.

Surface analysis using SEM revealed that only ST demonstrated mineral deposition after immersion in PBS and artificial saliva for 28 days. The cubical-like crystalline structures on ST’s surface suggest that it has some level of bioactivity, likely resulting from the ion-releasing capabilities of its unique fillers, such as calcium aluminate and ytterbium trifluoride. GICs and calcium aluminate fillers are known for their ability to release ions, which promote remineralization and form apatite-like deposition [31]. In particular, calcium aluminate has been shown to release calcium ions, which can promote the deposition of mineral phases resembling natural tooth enamel, thereby providing an additional layer of protection to the restoration [32]. Moreover, ytterbium trifluoride has been linked to enhanced bioactivity due to the release of fluoride, which plays a crucial role in enamel remineralization [33]. In contrast, CM and TE, which lack bioactive components, did not exhibit any surface changes after immersion in PBS and artificial saliva. This observation aligns with the findings of previous studies that emphasize the importance of bioactive fillers in facilitating ion release and subsequent mineral deposition on the surface of restorative materials. CM and TE are composed primarily of conventional inert glass/ceramic fillers with no specific bioactivity, which clarifies their inability to undergo surface changes during the immersion period. Without ion exchange or the release of specific ions such as calcium, phosphate, and/or fluoride, these materials cannot trigger remineralization processes, which is a key feature of bioactive materials in dental applications [34,35]. The observed mineral-like deposits on the surface of ST after immersion in phosphate-buffered saline and artificial saliva could be attributed to the unique chemical composition of the material, which may include functional monomers or reactive filler components capable of attracting calcium and phosphate ions. These ions can nucleate and grow into crystalline structures, suggesting potential bioactivity and the ability to support mineral deposition [35].

The composition of composite resins is complex. In this study, general information on the elemental structure of the evaluated composite resins was obtained using EDX [36,37,38]. EDX analysis further supported the SEM findings, as no significant compositional changes were detected in CM and TE over the 28-day period, indicating that these materials do not engage in ion exchange or mineral deposition.

In contrast, ST exhibited minimal yet noticeable compositional shifts that correlated with the mineral deposition observed through SEM, highlighting its potential bioactive nature. The interaction between ST and the surrounding environment appears to promote remineralization, which could have important clinical implications, such as enhancing the longevity and stability of dental restorations [19,20,39].

Wettability testing revealed that ST exhibited a significantly lower contact angle compared to CM and TE, indicating superior hydrophilicity. Hydrophilicity refers to the ability of a material to interact with water, which plays a critical role in determining how effectively a material can bond with the surrounding tissues and fluids. A lower contact angle means that the material has a stronger affinity for water, which can improve its ability to interact with substrates such as the dental tissues, thereby enhancing the adhesion and potentially increasing the clinical performance of the restoration over time [40]. Materials with lower contact angles tend to exhibit stronger surface interactions with water-based fluids, which can reduce the risk of debonding [41]. In the case of ST, it exhibits greater wettability compared to the conventionally tested composites. One possible explanation lies in the presence of GIC and Ca-aluminate fillers, which possess an affinity for water, making the material more hydrophilic [31,35].

Enhanced wettability is particularly important for materials with bioactive potential. When a material has excellent wettability, it is better equipped to facilitate mineral ion exchange with the surrounding environment, such as the release and uptake of calcium and phosphate ions. This ion exchange can contribute to the remineralization of the tooth structure and can aid in the formation of a protective layer on the surface of the material. Hydrophilic materials can enhance the process of remineralization by facilitating ionic interactions with the biological environment, making them ideal candidates for restorative materials in dental applications [42,43,44]. Bioactive materials that exhibit superior wettability, like ST, can release calcium ions and phosphate ions more efficiently, promoting the growth of hydroxyapatite on the surface of the material, which mimics a natural tooth enamel [20,34,35].

ST demonstrated comparable antibacterial performance to TE and the control group, while CM showed a significant reduction in bacterial growth. This observation suggests that CM may possess intrinsic antibacterial properties, potentially linked to additives in its formulation. Based on this, the present results seem in accordance with Ikeda et al., who observed resin composites with a higher filler content, which displayed a reduced biofilm retention [45,46,47]. In addition, this observation could also be related to the higher toxicity of the non-polymerized monomer of CM compared to other materials [48]. However, the antibacterial performance of ST could be further enhanced by incorporating antimicrobial agents, such as silver or zinc nanoparticles.

In terms of limitations, it is commonly known that in vitro research may not accurately capture the intricate dynamics of the oral environment, including pH variations and dynamic loads. Future research should focus on long-term clinical trials to validate these findings and explore the material’s performance in different restorative applications, including its interaction with various adhesive systems and its durability under thermal and mechanical cycling. The findings collectively highlight a link between the physicochemical properties and the mechanical performance of the tested materials. The lower porosity of ST, enhanced wettability, and bioactive mineral deposition contributed to its superior compressive strength and fracture resistance. These properties likely work synergistically, supporting ST as a robust alternative to conventional light-curing composites, particularly in clinical scenarios requiring durability and bioactivity.

## 5. Conclusions

In conclusion, the results of this study emphasize the potential of ST Automix as a robust restorative material alternative to amalgam, particularly in terms of compressive strength, reduced porosity, enhanced wettability, and bioactive properties. ST demonstrated higher compressive strength than TE and CM and was fractured in multiple smaller fragments compared to the brittle fractures of TE and CM. ST revealed cubical-like crystalline formations indicating some level of bioactivity, whereas no changes were observed for TE and CM. Future investigations should focus on long-term clinical evaluations and optimizing antibacterial performance to further expand its applications. In addition, further studies should be performed to evaluate the anti-biofilm properties of the tested polymers in the present study.

## Figures and Tables

**Table 1:** Chemical composition, manufacturers’ instructions, and lot number of the materials used.

Material	Chemical Composition	Manufacturer’ Instructions	Lot
Adhese Universal (Ivoclar Vivadent; Schaan, Lichtenstein)	MDP, bis-GMA, HEMA, MCAP, D3MA, ethanol, water, initiator, stabilizers, silicon dioxide	Just for etch-and-rinse procedure: Apply phosphoric acid gel onto the prepared enamel first, and then on to the dentin. The etchant should be left to react on the enamel for 15–30 s and dentin for 10–15 s. Then, rinse thoroughly with a vigorous stream of water for at least 5 s and dry with oil—and water -free compressed air until the etched enamel surfaces appear chalky white. Application of the adhesive. Starting with the enamel, completely coat the tooth surfaces to be treated with Adhese Universal. The adhesive must be scrubbed into the tooth surface for at least 20 s. This time must not be shortened. Applying the adhesive on the tooth surface without scrubbing is inadequate. Disperse Adhese Universal with oil—and moisture-free compressed air until a glossy, immobile film layer result. Important: Avoid pooling, since this can compromise the fitting accuracy of the permanent restoration. Light cure the adhesive for 10 s.	Z06NMY
Clearfil Universal Bond Quick (Kuraray Noritake, Tokyo, Japan)	Bis-GMA (10–25%), ethanol (10–25%), HEMA (2.5–10%), 10-MDP, hydrophilic amide monomer, colloidal silica, silane coupling agent, sodium fluoride, camphorquinone, water	Apply BOND with a rubbing motion to the entire cavity wall with the applicator brush. No waiting time is required. Dry the entire cavity wall sufficiently by blowing mild air for more than 5 s until BOND does not move. Use a vacuum aspirator to prevent BOND from scattering. Light-cure BOND with a dental curing unit (10 s or 5 s depending on the light type.	CK0443
Stela Primer (SDI Ltd., Bayswater, Victoria, Australia)	10-MDP, dimethacrylates, methyl ethyl ketone (MEK), water, initiators, stabilizers	Prime cavity and margins. Wait 5 s. Dry for 2–3 s.	1220447
Tetric EvoCeram A3 (Ivoclar Vivadent, Schaan, Liechtenstein) (TE)	Filler: 75–76 wt.% (53–55 vol.%) inorganic fillers; barium glass, ytterbium trifluoride, mixed oxide (particle size of the inorganic fillers 40 nm–3000 nm, mean size 550 nm) and prepolymer (34 wt.%) Matrix: Bis-GMA, UDMA, ethoxylated Bis-EMA	For optimum results, apply Tetric EvoCeram in layers of max 2 mm thickness or 1.5 mm thickness using Cavifil injector or a comparable applicator and adapt it with a suitable instrument. Adapt the material correctly to ensure intimate contact of the composite resin with the cavity walls. Prevent incomplete polymerization of the restoration by ensuring sufficient exposure to the curing light. For the recommendations regarding exposure time per increment and light intensity (20 s, 10 s, or 5 s depending on the light intensity).	Z071CG
Clearfil Majesty ES-2 A3 (Kuraray Noritake Dental; Tokyo, Japan) (CM)	Bis-GMA, Other methacrylic monomers, Surface treatment glass powder, Surface treatment organic composite filler, Photopolymerization catalyst, Pigments, etc.	Insert a pre-loaded tip into the dispenser barrel and extrude the paste into the dispenser barrel and extrude the paste into the cavity according to the instructions for use of the dispenser. Use the dispenser with a slow and steady pressure. Excessive force is not necessary. Discard the tip after use.	410173
Stela Automix (SDI Ltd., Bayswater, Victoria, Australia) (ST)	UDMA, GDMA, fumed silica, barium aluminoborosilicate glass, fluoro aminosilicate glass, ytterbium trifluoride (YbF_3_), calcium aluminate, hydroperoxide-based initiators, stabilizers, pigments	Stela: straight to placement in 15 s. Place Stela in a single increment, covering margins.	1226631

10-Methacryloyloxydecyl dihydrogen phosphate (10-MDP), Bisphenol A glycidyl methacrylate (Bis-GMA), Hydroxyethyl methacrylate (HEMA), Methacrylate copolymer (MCAP), Decanediol dimethacrylate (D3MA), Urethane dimethacrylate (UDMA), Ethoxylated bisphenol A dimethacrylate (Bis-EMA), Methyl ethyl ketone (MEK), Glycerol dimethacrylate (GDMA), Ytterbium trifluoride (YbF3), fumed silica (Amorphous silica nanoparticles), silane coupling agent (Silane-based adhesion promoter), and photopolymerization catalyst (Light-sensitive compound to initiate polymerization).

## Data Availability

The original contributions presented in this study are included in the article. Further inquiries can be directed to the corresponding author.
